# Effective Feature Engineering Framework for Securing MQTT Protocol in IoT Environments

**DOI:** 10.3390/s24061782

**Published:** 2024-03-10

**Authors:** Abdulelah Al Hanif, Mohammad Ilyas

**Affiliations:** Department of Electrical Engineering and Computer Science, Florida Atlantic University, 777 Glades Road, Boca Raton, FL 33431, USA; ilyas@fau.edu

**Keywords:** Message Queuing Telemetry Transport, Internet of things, security, machine learning, feature selection

## Abstract

The explosive growth of the domain of the Internet of things (IoT) network devices has resulted in unparalleled ease of productivity, convenience, and automation, with Message Queuing Telemetry Transport (MQTT) protocol being widely recognized as an essential communication standard in IoT environments. MQTT enables fast and lightweight communication between IoT devices to facilitate data exchange, but this flexibility also exposes MQTT to significant security vulnerabilities and challenges that demand highly robust security. This paper aims to enhance the detection efficiency of an MQTT traffic intrusion detection system (IDS). Our proposed approach includes the development of a binary balanced MQTT dataset with an effective feature engineering and machine learning framework to enhance the security of MQTT traffic. Our feature selection analysis and comparison demonstrates that selecting a 10-feature model provides the highest effectiveness, as it shows significant advantages in terms of constant accuracy and superior training and testing times across all models. The results of this study show that the framework has the capability to enhance the efficiency of an IDS for MQTT traffic, with more than 96% accuracy, precision, recall, F1-score, and ROC, and it outperformed the most recent study that used the same dataset.

## 1. Introduction

The rapid expansion of IoT devices has radically changed technology in recent years. Devices’ ability to connect and exchange data without requiring human interaction can enhance the daily activities of people and businesses and drive innovation [1]. These IoT devices are prevalent in diverse domains, including health care, smart cities, energy, smart homes, and industry. However, the IoT’s rapid development has resulted in potential areas of attack and created attractive targets in the form of weaknesses or vulnerabilities in the system [2,3,4]. Breaches in the security of IoT devices and networks can lead to disruption of system infrastructure, privacy violations, and other issues. IoT communication device systems must be secure and able to execute different security measures to prevent possible attacks.

In the IoT, to facilitate secure and dependable data transmission, various communication and messaging protocols have been created, such as Extensible Messaging Presence Protocol (XMPP), Constrained Application Protocol (CoAP), Message Queuing Telemetry Transport (MQTT), and Advanced Message Queuing Protocol (AMQP) [5]. These protocols are used for different situations; in particular, MQTT has been utilized in healthcare monitoring, smart agriculture, smart homes, and industrial applications due to its lightweight messaging protocol and efficient communication between machine-to-machine devices [6]. However, using the MQTT protocol in the IoT without implementing adequate security measures opens up a number of cybersecurity threats.

To protect and improve data exchange with high network security, an intrusion detection system (IDS) is a widely acknowledged approach for identifying and mitigating network intrusions. An IDS is crucial for security in IoT environments and enables monitoring and protection of IoT networks, detection of suspicious activity, and identification of threats [7]. IDSs utilize various methods, such as machine learning (ML), to detect and categorize possible threats to a secure network’s traffic [8]. Integrating ML techniques into an IDS effectively enhances the security of MQTT networks.

The rapid evolution of the IoT has resulted in an increasing need for efficient AI and ML methods such as tiny ML, microcontroller-based ML, and deep learning to improve security and mitigate malicious attacks. These technological advances allow for the direct implementation of ML on IoT devices and minimize complexity while detecting and addressing threats in real-time [9].

ML is efficient in various application areas and is considered a thriving AI method that can automatically learn significant knowledge from extensive datasets [10]. ML can achieve excellent detection results and display powerful generalization abilities with sufficient and well-prepared training datasets [11]. ML provides a practical approach to improving IoT security through the utilization of various supervised, unsupervised, and reinforcement learning methods [12]. Due to their strategy of effectively identifying distinctions between normal and abnormal data with high accuracy, ML methods have a robust advantage in intrusion detection systems [13].

While the MQTT protocol is well-known for its lightweight and efficient nature, it also presents vulnerabilities in secure implementations because it lacks encryption and authentication mechanisms [14]. For instance, an attacker could exploit protocol weaknesses to launch denial-of-service (DoS) attacks on the server or compromise devices to gain unauthorized access to sensitive data stored on the server. Attackers can also intercept and monitor user chat message communications, violating privacy and confidentiality. Consequently, developing an IDS to mitigate malicious attacks on the MQTT protocol is essential. Recent research has explored multiple ML techniques for intrusion detection, but some of these techniques still need to improve their ability to detect attacks [15]. A large dataset is essential to effectively train an ML model to detect legitimate and malicious behaviors. However, the large amount of data generated by IoT devices creates a challenge for resource management. The Authors of [10,16] have proved that the use of feature engineering techniques can improve intrusion detection accuracy by focusing on essential features during model training and tuning while reducing the dataset size. Utilizing optimal input feature engineering can significantly enhance the detection accuracy and effectiveness of a model [17]. The feature engineering technique has become an essential phase during a system’s overall processing [18]. Using feature selection in feature engineering is a vital step that enhances the overall efficacy of ML algorithms. The feature selection strategy can balance high classification performance with resource optimization, resulting in high efficiency and making it an attractive option for intrusion detection in an IoT network environment.

This study presents a solution for developing an effective IDS for MQTT traffic through binary classification. This approach involves employing designed feature engineering and optimized ML models to detect and classify IoT network traffic as normal or abnormal. The major contributions of this study are:

- An approach to feature subset selection that can select the imperative features in the dataset, including the Pearson coefficient correlation (PCC), ExtraTreesClassifier, and RandomForestClassifier.
- Applying an analysis of all 33 features in the dataset by comparing subsets of 33 to 20, 15, and 10 features, respectively, to determine the most critical features.
- Providing a novel approach by selecting the top 10 features to sustain high performance in classification and reduce the dimensionality of the feature set. This leads to results that have several advantages, including making the model faster at training and testing, making it easier to interpret intrusion detection, improving accuracy, concentrating on the most necessary information while minimizing the effect of extraneous factors; and being more efficient with less storage and processing.
- The evaluation of several ML methods to select the most appropriate models for accurately and efficiently constructing an effective IDS to identify attacks on IoT MQTT traffic. This involves applying cross-validation and hyperparameter optimization for ML models. The evaluated models include Decision Tree (DT), Random Forest (RF), k-nearest Neighbor (KNN), Adaptive Boosting (AdaBoost), and Xtreme Gradient Boosting (XGBoost) classifiers. Evaluation metrics include accuracy, precision, recall, F1-score, and area under the curve receiver operator characteristic (ROC-AUC).
- A comparison between our model and recently proposed models using the same dataset. Our model demonstrates superior performance in detecting attacks.

This paper is arranged in the following way: Section 1 is the introduction. Section 2 summarizes related work. Section 3 provides an overview of the MQTT protocol’s background and discusses its many attack types. Section 4 explains the methods and materials utilized in this study. Section 5 presents the findings, comparisons, analysis, and discussion. Finally, Section 6 is the conclusion of this paper.

## 2. Related Work

The domain of IoT security is an active research area that is attracting significant attention from scholars worldwide. The rapid growth of IoT devices has resulted in various attacks, making IoT networks vulnerable to multiple threats. This section briefly reviews recent advancements in enhancing IoT security, focusing on MQTT protocol targeted approaches for the IoT. Table 1 summarizes the related work of recent research.

Alaiz-Moreton et al. [19] focused on improving the identification of attacks on the MQTT-IoT protocol by using a multiclass classification method. The authors used multiple approaches, including XGBoost, long short-term memory (LSTM), and GRU, to distinguish between normal traffic and attacks on the IoT system. Their dataset included various types of attacks such as man-in-the-middle (MitM), denial-of-service (DoS), and intrusion. They found that these methods demonstrated excellent efficiency in attack detection, with XGBoost models exhibiting higher accuracies compared to LSTM and GRU. However, LSTM and GRU models outperformed linear models.

Ciklabakkal et al. [20] presented a framework for a lightweight anomaly-based IDS for IoT environments, with a primary focus on MQTT assaults. They employed an effective ML method that included an autoencoder, single-objective generative adversarial active learning (SO GAAL), RF, isolation forest, one-class support vector machines (OCSVM), and K-means to detect and mitigate anomalies in the MQTT protocol. This system was designed to identify the type of attack and generate alerts upon detecting anomalies. The study showcased the ability of ML techniques to detect attacks even without prior knowledge of the types of anomalies. However, the authors needed to employ another method to achieve high accuracy.

Hinde et al. [21] proposed a study that depended on IDS ML for MQTT networks. They constructed a new MQTT dataset containing benign and attack situations. The authors chose and evaluated six distinct ML methods: k-NN, LR, DT, naive Bayes (NB), support vector machine (SVM), and RF regarding precision, recall, and F1-scores. According to the study results, flow- and packet-based characteristics are essential for identifying malicious attacks. Flow-based features examine strategies for mitigating and identifying MQTT-based malicious attacks as well as packet-based features associated with traditional networking attacks. The authors also highlighted the requirements and challenges of constructing an IDS for the IoT based on the MQTT protocol due to its particular communication patterns.

Vaccari et al. [22] introduced the MQTTset dataset, which focuses on the MQTT protocol in IoT networks. This dataset comprises two types of traffic—legitimate and malicious traffic—and can be utilized to detect anomalies in IoT system environments by training ML models. The research team employed a balanced method to evaluate the dataset and executed a diverse set of ML algorithms, including NB, MLP, RF, GB, NN, and DT. They achieved more accurate results by using the balanced dataset, evidenced by high accuracy and F1-scores.

Khan et al. [6] presented a deep-learning-technique-based IDS for the MQTT protocol in the IoT. They evaluated the performance of the model by comparing a deep neural network (DNN) model with base ML methods, including RF, DT, LSTM, NB, gated recurrent units (GRUs), and KNN, using two different datasets: MQTT-IoT-IDS2020 and a dataset containing three different attack types. According to the results, the DNN model achieved high accuracies for both scenarios and surpassed other ML methods. This article does not analyze different kinds of attacks; it primarily emphasizes evaluating the DNN method.

Makhija et al. [23] utilized ML techniques such as the KNN, RF, and SVM classifiers to predict the efficiency of how datasets can be exploited in an MQTT-based IoT environment. The authors also evaluated the ML techniques by using accuracy and F1-scores to determine the detection systems’ effectiveness. The experiment’s results demonstrate that RF achieved higher accuracy than the other methods. However, in this study, the researchers did not utilize the approach of oversampling and feature selection, and the RF classifier had high accuracy because it only identified one strike.

Vijayan et al. [24] aimed to detect attacks in IoT environments by proposing an intrusion detection model based on the CatBoost classifier model for the MQTT protocol. The authors employed multiclass classification to categorize the type of attack and trained the data on both balanced and imbalanced traffic MQTT datasets. They found the CatBoost method to be superior to traditional ML algorithms such as Neural Network (NN), RF, NB, DT, Gradient Boost (GB), and MLP. It achieved high accuracy, precision, recall, and F measure for both scenarios. Nonetheless, the paper needed to enhance the performance of model detection.

Siddharthan et al. [16] proposed the SENMQTT-SET framework as an IDS system for serving the environment of IoT-MQTT networks. They executed experiments using Raspberry Pi and NodeMcu devices linked to a router, and they created three different types of scenarios: normal behavior, attacks targeting a subscriber, and attacks targeting a broker. The authors utilized elite ML algorithms (EML) to select the most appropriate model for intrusion detection from a range of ML algorithms, such as SVM, KNN, DT, RF, GB, NB, and logistic regression (LR). They used F1-score, accuracy, Matthews correlation coefficient (MCC), receiver operating characteristic (ROC), false alarm rate (FAR), detection rate (ADR), negative predictive value (NPV), and positive predictive value (PPV) to evaluate the performance metrics. The DT algorithm got better results for training time and detection time while exhibiting lower scores in other performance metrics compared to other algorithms. This experiment needed to improve multi-modal traffic classifiers by using different methods such as deep learning.

Zeghida et al. [25] proposed ensemble learning for an intrusion detection model based on the MQTT protocol to enhance the IoT security environment. They discussed utilizing multiple ML methods to detect intrusion in the MQTT IoT structure. The researchers evaluated and compared all the different models used in the paper and found that the stacking model of ensemble learning approaches offered higher improvement in terms of accuracy, F1-score, and MCC.

Muñoz Castañeda et al. [26] focused on using ML methods to identify intrusions in IoT environments based on the MQTT protocol and other attacks. The authors proposed a hybrid feature selection technique to effectively determine the type of traffic (normal vs. attack) while also explaining the underlying features. The algorithm successfully identified features that indicated distinct types of IoT attacks. They also discussed the significance of utilizing entropy-based methods for feature selection to detect IoT attacks, but this experiment needed more training to develop ML classifiers.

In this study, we propose a solution to construct an efficient IDS framework for MQTT traffic using feature engineering and optimized ML models. Analyzing all 33 features in the dataset and comparing subsets of 33, 20, 15, and 10 features showed that the 10-feature selection provides notable benefits, including decreased processing time throughout both the training and testing phases and consistent accuracy across all models. However, it is important to acknowledge potential limitations, such as the possibility of losing information when reducing features. Therefore, it is necessary to rely on feature selection approaches and thoroughly evaluate model generalization. Table 2 compares our approach and recently proposed approaches regarding feature selection, data balancing, hyperparameter tuning, cross-validation, and accuracy.

In Table 2, “+” denotes the utilization of a method and its subsequent recognition as a strength, while a “−” denotes the non-utilization of a method and its consequent identification as a weakness.

## 3. Background of MQTT Protocol

### 3.1. Overview

In 1999, the MQTT protocol was invented by Andy Stanford-Clark and Arlen Nipper. In 2013, it became an accepted standard by the Advancement of Structured Information Standards (OASIS) [27].

MQTT is widely recognized to be highly beneficial for machine-to-machine and IoT applications. It runs on the top layers of TCP/IP, which is the application layer protocol [27]. The design principle of the MQTT protocol is to transfer data while maintaining reliable delivery [1]. The MQTT protocol is able to work in different domains because it has several benefits, such as low packet loss, low bandwidth, and low memory requirements.

**Figure 1:**
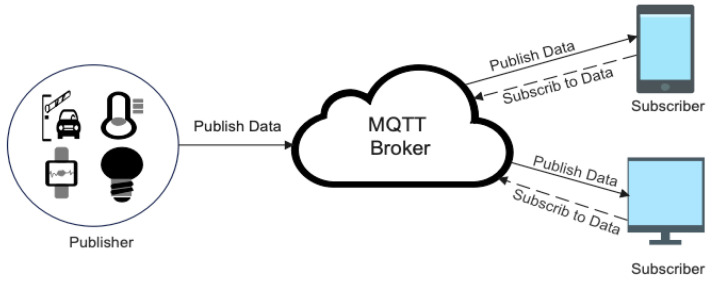
Structure of MQTT publish/subscribe.

MQTT is a communications protocol based on three components: subscribers, publishers, and brokers. Figure 1 demonstrates the MQTT publisher/subscriber structure. MQTT depends on the publish–subscribe model, whereby clients create connections with a broker, and afterwards, messages are distributed when they are published on certain topics [28].

MQTT messaging works as follows:

- The publisher sends a message about a specified MQTT topic via the broker.
- After receiving publisher messages, the broker verifies its subscriber list to determine who is related to this topic and then forwards the messages to whomever is interested.
- After subscribers get the messages, they process them depending on their subscriptions’ relevance.

### 3.2. MQTT Security

The MQTT protocol has a significant role in facilitating communication within the IoT ecosystem. The primary goals of the MQTT protocol are to consume little energy, be lightweight, and to optimize bandwidth without prioritizing security considerations. As a result, the MQTT system can be vulnerable to various security attacks and threats that might endanger the safety and integrity of data traveling across the MQTT network [29]. Some common MQTT attacks include DoS attacks, message spoofing, eavesdropping, and MitM attacks. Securing MQTT protocol communications and implementing additional security measures is necessary to prevent and control security attacks and threats in the network.

### 3.3. MQTT Threats

The MQTT protocol in the IoT field is susceptible to multiple threats due to its inherent weaknesses. Below is a list of attacks targeting the MQTT protocol and which are part of the MQTTset dataset used in the paper.

Denial-of-Service (DoS) Attack: This type of attack disrupts the network system’s ability to provide services to authorized users [27]. MQTT brokers are vulnerable to attacks that can consume bandwidth by transferring large packets that exceed the MQTT payload size and by sending large numbers of messages with a quality of service level 2 [30].

Brute-Force Attack: The impact of this type of attack on IoT networks has become increasingly prevalent and results in significant harmful consequences [31]. During the authentication process, a brute-force attack aims to decipher the authentication credentials of users’ information, such as usernames and passwords [32].

Malformed Attack: The MQTT protocol is vulnerable to exploitation and targeting of specific categories of single-packet attacks known as malformed attacks. This type of attack aims to produce and send many inaccurate, malformed packets to the broker to trigger exceptions within the determined service [32].

SlowITe Attack: This is considered a new type of DoS that targets the MQTT protocol. This type of slow DoS attack utilizes minimal attack bandwidth and exhausts server resources to disrupt the functionality of a network service through a denial of service [33].

Flood Attack: This type of attack can be employed against the MQTT protocol and different network services. Its purpose is to obstruct the creation of services from law-abiding customers [32].

## 4. Methods and Materials

### 4.1. Proposed Approach

This paper proposes a binary classification task on the MQTT protocol of network traffic data that seeks to classify the traffic into two categories: normal and abnormal. This proposal aims to construct and improve the MQTT protocol’s IDS efficiency by employing feature selection algorithms and then utilizing ML models to detect attacks on IoT devices. RF, DT, KNN, AdaBoost, and XGBoost are the ML models utilized in this study. This work also investigates which features help detect the most malicious activity on the system. Then, we choose the most relevant features to improve results and reduce overfitting, particularly in cases with redundant or irrelevant features. Additionally, hyperparameter optimization is used, which helps ensure that all the model classifiers are being fine-tuned to achieve better performance by using the grid-search algorithm. This performance evaluation includes critical metrics such as accuracy, precision, recall, F1-score, and ROC-AUC.

### 4.2. Environment Tools

In this study, a Jupyter Notebook was used to implement and develop the proposed model that the Anaconda platform provides. It is considered a highly robust and interactive computational environment, and it is widely utilized in the field of artificial intelligence (AI) [34]. The Python programming language was chosen for this study due to its frequent use as the language of choice for ML libraries and its multiple advantages in terms of effectiveness, stability, evaluation, and scalability.

### 4.3. Workflow of MQTT Network Traffic with ML

**Figure 2:**
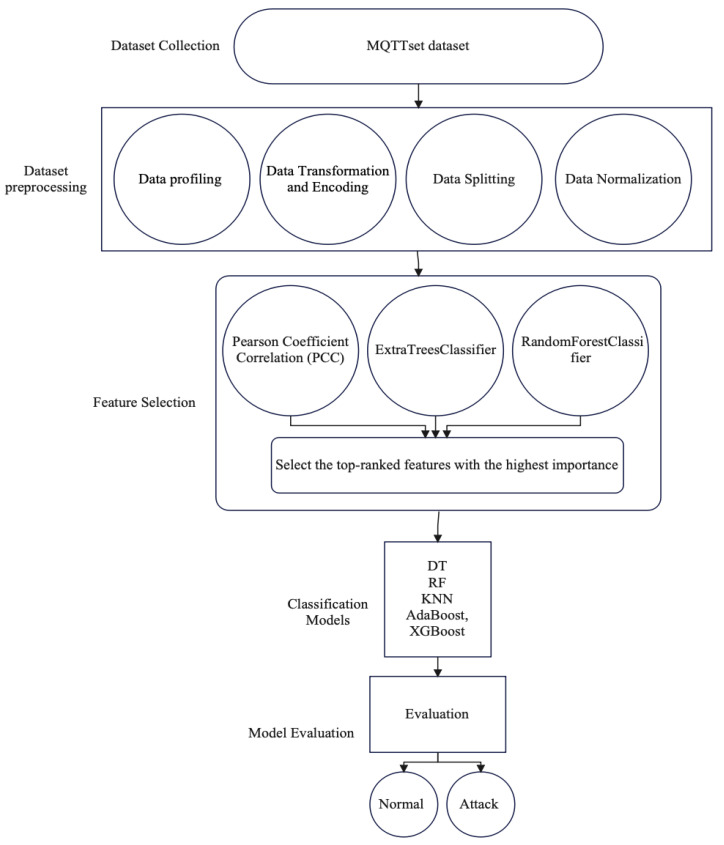
The workflow of MQTT network traffic associated with the utilization of ML models.

Figure 2 depicts the workflow for the MQTTset IDS dataset based on ML techniques. It begins with the collection of data, including the dataset of MQTT network traffic information. Then, dataset preprocessing is performed, which includes multiple steps such as data profiling to get insights into the dataset characteristics, data encoding, and transformation techniques. This prepares the dataset for modeling, which involves data splitting to create training and testing sets and data normalization for ensuring the dataset of feature scales remains constant. Next, feature selection methods such as PCC, ExtraTreesClassifier, and RandomForestClassifier are applied to determine the most informative features. After that, the preprocessed data are utilized to train multiple classification models. Finally, performance metrics are used to evaluate the model.

### 4.4. Data Collection

#### MQTTset Dataset

The MQTTset dataset utilized in this paper was created by Vaccari et al. [22]. It was made available to the public in 2022 through the Kaggle enterprise. This dataset serves as a significant and valuable resource for IDS research in the IoT field. It particularly concentrates on developing detection systems for the MQTT protocol within IoT networks. The dataset scenario is made within a smart home environment whereby eight MQTT sensors are used to collect various data types such as humidity, CO gas, light intensity, temperature, smoke, motion sensor, fan sensor, door lock, fan, and speed controller. The transmitted readings of the data of these sensors arrive at varying temporal intervals due to the different behavior of each sensor. Table 3 of the MQTTset dataset includes legitimate and malicious attack traffic involving legitimate, DoS, malformed, slowite, brute-force, and flood attacks.

### 4.5. Dataset Preprocessing

#### 4.5.1. Data Profiling

The initial step in data preprocessing seeks to understand and describe the raw data before starting modeling, analysis, and other related methods. In this phase, we conducted a comprehensive examination to produce statistical information properties, data types, missing values, balancing techniques, insignificant features, and empty values. This examination aimed to determine whether the data were numerical or not and to check if normalization was necessary. We used the MQTTset dataset reduced version available at Kaggle by Vaccari et al. [22], from which we obtained balanced representational data and cleaned data regarding missing values, extracted values, and quality.

#### 4.5.2. Data Transformation and Encoding

Label encoding is significant for the ML method, as it aims to transform categorical data into numerical values. This approach includes assigning a distinct integer to the unique class or category that exists inside a categorical variable. It was a suitable choice for this study due to its ability to convert to numerical values to avoid increasing the number of features or the computational complexity of the modeling method. We applied label encoding on multiple columns of the dataset, such as ‘tcpṫime_delta’, ‘mqttċonack.flags’, and ‘tcpḟlags’, to convert their categorical values to a numeric form. As our approach in this study focuses on the differentiation between normal and abnormal network traffic, we used the encoder function (lambda) to effectively decrease the number of output targets to make it binary as 0 (legitimate data) and 1 (attack data).

#### 4.5.3. Data Splitting

Splitting the dataset is significant for using ML to develop and evaluate the model. It is also considered robust for building intrusion detection models capable of distinguishing between normal and abnormal network traffic. The dataset is divided into training and testing sets, with 70% being the training set and 30% being the test set.

#### 4.5.4. Data Normalization

Data normalization is a significant step in preprocessing data. It aims to standardize the data while maintaining the disparities in the ranges of each specific feature. It is also essential to use normalization when the features have different scales on the dataset, as it assists in enhancing ML performance. This study used min–max scaling to standardize each feature, which was applied to training and testing data using fit_transform. The min–max scaling technique transformed each feature to a predetermined range, often between 0 and 1 [35]. It was performed by subtracting the feature’s minimum value from each data point and dividing by the range, which is defined as the difference between the maximum and minimum values [35]. The formula for min–max scaling is as follows:

(1) X_normalized=[X−min(X)]/range(X),X_normalized=[X−min(X)]/[max(X)−min(X)]

where: *X_normalized* is the scaled value, *X* is the original data point, *min(X)* is the minimum variable for features inside the dataset, and *max(X)* is the maximum variable for features inside the dataset.

### 4.6. Feature Selection

Feature selection algorithms and dimensional reduction techniques were utilized to analyze datasets by using statistical approaches, ML methods, and information theory to improve the IDS and decrease the complexity of the IDS model [16]. Feature selection is an essential process in ML that involves concentrating on selecting a subset of the most valuable, relevant, and informative features from a dataset and eliminating others that are irrelevant or less important. Choosing the most important features enhances the model’s performance, decreases overfitting, and streamlines the modeling process [10]. PCC, ExtraTreesClassifier, and RandomForestClassifier are common feature selection techniques used in IDS models and were selected for this work. We performed an analysis of these methods to identify the most significant feature within MQTT traffic for an accurate classification result.

#### 4.6.1. Pearson Coefficient Correlation (PCC)

**Figure 3:**
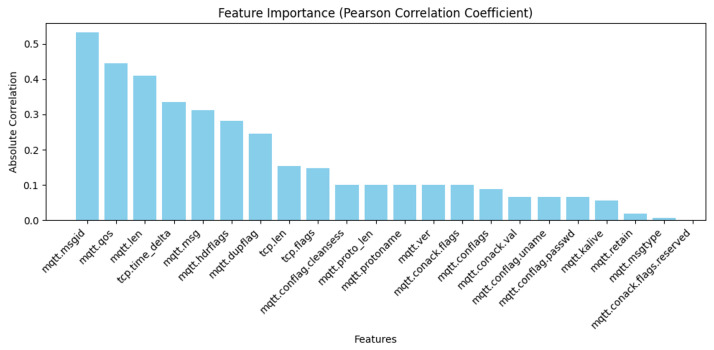
Feature selection using PCC.

PCC is used to identify the most relevant features and is considered standard for calculating the statistics between two different features. The range of this value falls between [−1, 1], and the relationship of the PCC value sign between the variables might be positive or negative [36]. Figure 3 shows the results for these features.

#### 4.6.2. ExtraTreesClassifier

**Figure 4:**
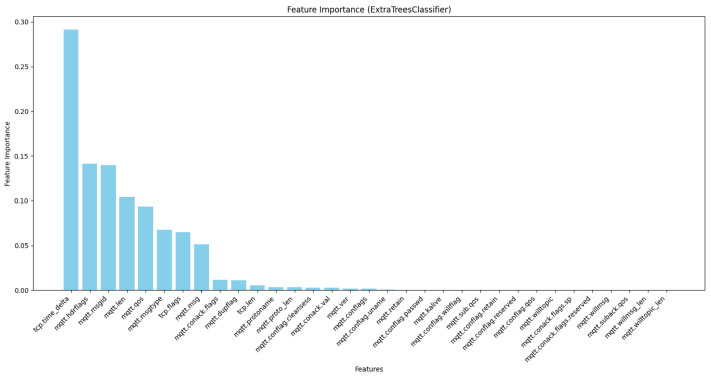
Feature selection using ExtraTreesClassifier.

This type of feature selection refers to an ensemble method to construct numerous decision trees and aggregate their predictions to increase the level of outcome accuracy and produce more vital predictions [37]. In this study, ExtraTreesClassifier was run on the dataset to determine the most significant feature. Figure 4 shows the result for these features.

#### 4.6.3. RandomForestClassifier

**Figure 5:**
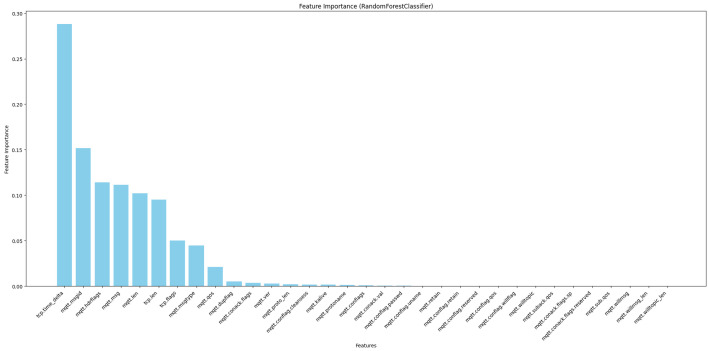
Feature selection of RandomForestClassifier.

RandomForestClassifier is used to select the most significant features concerning the target variable [38]. This study used this technique to select the best features, as illustrated in Figure 5.

After performing all the aforementioned methods, we selected the final set of features based on the highest scores achieved, which all methods reached a consensus on. Table 4 displays the final set selection along with its description.

### 4.7. Classification Models

Classification models refer to types of ML models that are utilized to predict categorical labels. The purpose of these models is to assign labels to input data based on learned system patterns and behavior from predefined training data; thus, ML methods assist the IDS with detecting and analyzing types of security threats as normal or malicious. In this study, we evaluated multiple ML methods to determine the most valuable and accurate classifier for building a robust MQTT protocol IDS. We used five methods: RF, DT, KNN, AdaBoost, and XGBoost. We used feature selection, as shown in Table 4, to determine the output types as either normal or malicious, with 0 indicating a normal attack and 1 indicating an abnormal attack.

## 5. Results

### 5.1. Performance Metrics

Our scheme used the evaluation metrics accuracy, precision, recall, F1-score, and ROC to evaluate the performance of the strengths and weaknesses of classification models. The evaluation metrics for performance are obtained from the confusion matrix utilizing Equations (2)–(7) [39]. Each equation has a specific role in evaluating the intrusion detection system’s effectiveness. The terms are defined as follows:

- True positive (TP): represents the number of times our model correctly classifies positive MQTT network traffic instances as positive attacks [40].
- False positive (FP): represents the number of times our model incorrectly classifies negative MQTT network traffic instances as positive attacks [41].
- True negative (TN): represents the number of times our model correctly classifies negative MQTT network traffic instances as normal attacks [40].
- False negative (FN): represents the number of times our model incorrectly classifies positive MQTT network traffic instances as normal attacks [41].

#### 5.1.1. Accuracy

Accuracy is an evaluation metric utilized to assess classification models; it represents the ratio of correctly predicted samples to the total number of samples [42]. The calculation of the mathematical equation is represented as follows:

(2) Accuracy=(TP+TN)/(TP+TN+FP+FN)

#### 5.1.2. Precision

Precision measures the number of malicious samples of true positive predictions divided by all samples predicted as positive. The calculation of the mathematical equation is represented as follows [41]:

(3) Precision=TP/(TP+FP)

#### 5.1.3. Recall

Recall defines the percentage of true positive predictions among the total actual number of positive instances. The calculation of the mathematical equation is represented as follows [42]:

(4) Recall=TP/(TP+FN)

#### 5.1.4. F1-score

F1-score is defined as the weighted average of precision and recall, and it is calculated using the mean of the harmonic. The calculation of the mathematical equation is represented as follows [41]:

(5) F1−Score=2∗(Precision∗Recall)/(Precision+Recall)

#### 5.1.5. ROC-AUC

ROC-AUC is used to distinguish between positive and negative classes. The ROC curve represents the difference between the true positive rate and the false positive rate when the different classification threshold values are altered. The calculation of the mathematical equation of TPR and FPR is represented as follows [43]:

(6) TruePositiveRate(TPR):TPR=TP/(TP+FN)

where:

(7) FalsePositiveRate(FPR):FPR=FP/(FP+TN)

### 5.2. Results and Discussion

#### 5.2.1. Hyperparameter Tuning

Hyperparameter tuning is a crucial step in an ML model to enhance model performance and provide a fair basis for comparing various models. Most researchers agree that tuning the hyperparameters of ML algorithms results in a more effective model and improves accuracy [44]. Hyperparameters are adjustable values or weights that influence the learning process of an algorithm and prediction processes for optimal performance. These hyperparameters are set before the training process on the model. In this study, we used a grid search to get the optimal set of hyperparameters for improved accuracy. Table 5 summarizes the optimized hyperparameters utilized in this study for each model.

#### 5.2.2. Performance Analysis

In this study, we conducted a feature importance analysis for MQTT traffic by using three different models: PCC, ExtraTreesClassifier, and RandomForestClassifier. According to the results obtained from these three models, we constructed Figure 3, Figure 4 and Figure 5; our approach depends on these figures to select the essential features. We used four steps to analyze the features. The first step was choosing all 33 features in the dataset to understand their contributions to the decision-making process. In the second step, we chose the highest 20 features based on their importance rankings from the three models. In the third step, we reduced the feature set to the top 15 features. In the last step, we chose the top 10 features of significant relevance in all models. These steps help to prioritize features based on their essential scores from three models to improve the efficiency and interpretability of our models.

Based on Figure 3, Figure 4 and Figure 5, we observed that only 17 to 21 features from a total of 33 features had an apparent effect on the decision-making process, while the remaining features had a feature relevance score of zero. We evaluated some of the metrics with model training using RF, DT, KNN, AdaBoost, and XGBoost on all four steps by using a hyperparameter with all features. We noticed that the accuracy improved compared with other studies, but when reducing features, the model’s overall performance maintained nearly consistent accuracy and, and we got a better accuracy result when using the top 10 selected features for some ML techniques. The main observation is that reducing the feature dimensionality can make the model faster in training and testing, as shown in Table 6, Table 7, Table 8 and Table 9. It also results in making ML models more effective while enhancing accuracy. The utilization of feature selection and the hyperparameter optimization strategy lead to efficiently enhanced results and accurate cyber-attack detection.

Table 6, Table 7, Table 8 and Table 9 illustrate the results of applying the four steps divided based on the PCC, ExtraTreesClassifier, and RandomForestClassifier models. These tables also illustrate the evaluation metrics (accuracy, precision, recall, F1-score, and ROC-AUC) of the classification of each tuned model, which shows that ML methods deliver high accuracy for all models. As observed in all tables, the training and testing times decrease with the reduction in feature selection. The accuracy maintains a consistently high level in all tables, showing relatively small variations and better accuracy results in Table 9. The RF method is considered the best-performing technique in all tables for accuracy and F1-score. The evaluation metrics F1-score, precision, and recall are also considered stable, which indicates the robustness of the models for classifying data points.

In this study, all experiments were performed several times and with five-fold cross-validation. This helped evaluate the overall quality and reliability of models and may disclose elements not clearly visible during the initial training stage.

A comprehensive analysis of the results indicates that researchers should focus on the 10 features that offer the highest values of feature importance to get sufficient accuracy: namely, [‘mqtt.qos’, ‘mqtt.msgid’, ‘mqtt.len’, ‘tcp.time_delta’, ‘mqtt.msg’, ‘mqtt.hdrflags’, ‘mqtt.dupflag’, ‘tcp.len’, ‘tcp.flags’, ‘mqtt.conack.flags’]. These selected features can achieve high performance in detecting cyber attacks on MQTT traffic. They are also considered to be better than the full feature set or subset (20 features and 15 features, respectively) in terms of training time and testing time and improve the overall accuracy. We also noticed that these ten features influenced the model’s accuracy. Deleting one of these features results in a decrease in accuracy, implying that a particular feature significantly contributes to enhancing the model’s capacity to identify and analyze patterns or attributes within the dataset.

**Figure 6:**
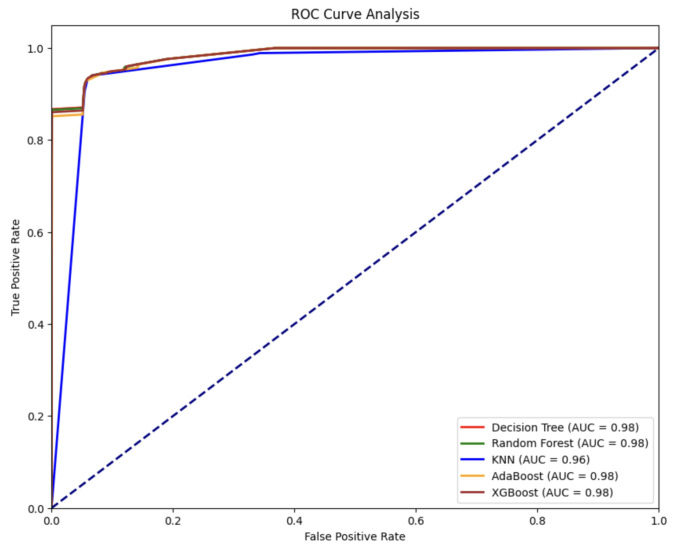
ROC results for ML algorithms.

Table 9 shows the results of the final set of 10 features with various ML techniques. The results demonstrate that RF achieved the highest accuracy (0.9633) and F1-score (0.9632) among the evaluated models. DT, KNN, and XGBoost demonstrate significantly shorter training periods than RF and AdaBoost, and RF and AdaBoost have relatively longer training times. During the testing time, DT and XGBoost provided faster model evaluation compared to the other models. The evaluation metric of ROC scores is generally high for all methods, but XGBoost achieved the highest ROC score (0.9847). Figure 6 depicts the ROC findings of the developed ML algorithms.

ML algorithms significantly enhance the accuracy and effectiveness of a model. ML techniques have a particular set of strengths, weaknesses, and suitability of algorithms. To select the proper ML method, the characteristics of the data should be understood. In this study, we examined multiple ML methods to select the appropriate models to develop an effective IDS to detect attacks on MQTT traffic. The DT method was selected based on its interpretability, simplicity, and ability to handle non-linear correlations in the data [45]. The KNN algorithm was chosen because of its simplicity and ease of implementation [46]. RF and XGBoost were chosen due to their popularity in ML methods and their ensemble learning capabilities that improve predictions by integrating numerous weak learners [45]. The AdaBoost method is a popular choice in various ML applications due to its applicability in different ML tasks and lower susceptibility to overfitting than other algorithms [45]. Also, tuning the hyperparameters for these models made a significant impact on the model performance. Reducing feature selection also helps improve the model’s performance with regard to training time, testing time, and accuracy.

Table 10 shows the results of papers [22,25] and compares our proposed method using the following evaluation metrics: accuracy, precision, recall, F1-score, ROC, and performance time for each chosen approach.

As shown in Table 10, paper [22] uses 33 features, and paper [25] uses 31; our model uses 10 features. This indicates that our scheme focused on the top 10 features for intrusion detection, maintained high classification accuracy of the model, and has superior performance in all evaluation metrics.

The main observation is that our approach significantly reduced training and testing times, while [22,25] have longer times; therefore, our method is better suited for applications that require real-time processing. It is also better at optimizing resources, improving efficiency, responding faster to preventing or underestimating the impact of attacks, and for scalability of intrusion detection models.

Compared to other studies, our scheme is also better at various other factors, including dataset preprocessing, data balancing, feature selection, hyperparameter optimization, and cross-validation. These elements make our approach a robust and effective solution for intrusion detection in the MQTT IoT traffic network. Paper [25] achieved better results in accuracy than paper [22] using all the ML methods. The authors did not use hyperparameter tuning in paper [22], while this was not reported for some MLs in [25]. Additionally, neither paper employed cross-validation in their methodologies.

Using many feature sets without clear selection criteria raises questions regarding the significance and effectiveness of the chosen features. It also might result in problems such as overfitting and interoperability. Reducing the number of features has several advantages in simplicity and by removing irrelevant features. However, this reduction may also result in disadvantages, such as losing valuable information [47]. Various techniques can be employed to avoid and mitigate this loss when reducing features. Employing several selection techniques rather than relying on a single feature selection method helps to explore and compare essential features within the dataset. In our research, we utilized three techniques—PCC, ExtraTreesClassifier, and RandomForestClassifier—to ensure that the chosen features have reliable and consistent selection across different methods. According to Table 10, paper [22] utilized a 33-feature set, while paper [25] used a 31-feature set, indicating they did not use feature selection. In contrast, we analyzed and compared to decrease the number of features in the set based on the feature selection techniques of PCC, ExtraTreesClassifier, and RandomForestClassifier for all 33 features. After that, our proposed model depends on the ten selection features to improve the reliability of the IDS of MQTT traffic and other factors such as thorough preprocessing, hyperparameter tuning, the optimal selection of ML models, data balancing, and applied cross-validation. This leads to several advantages, such as improving the model accuracy, reducing dimensionality, and facilitating faster testing and training for models compared to other studies, as shown in Table 10.

**Figure 7:**
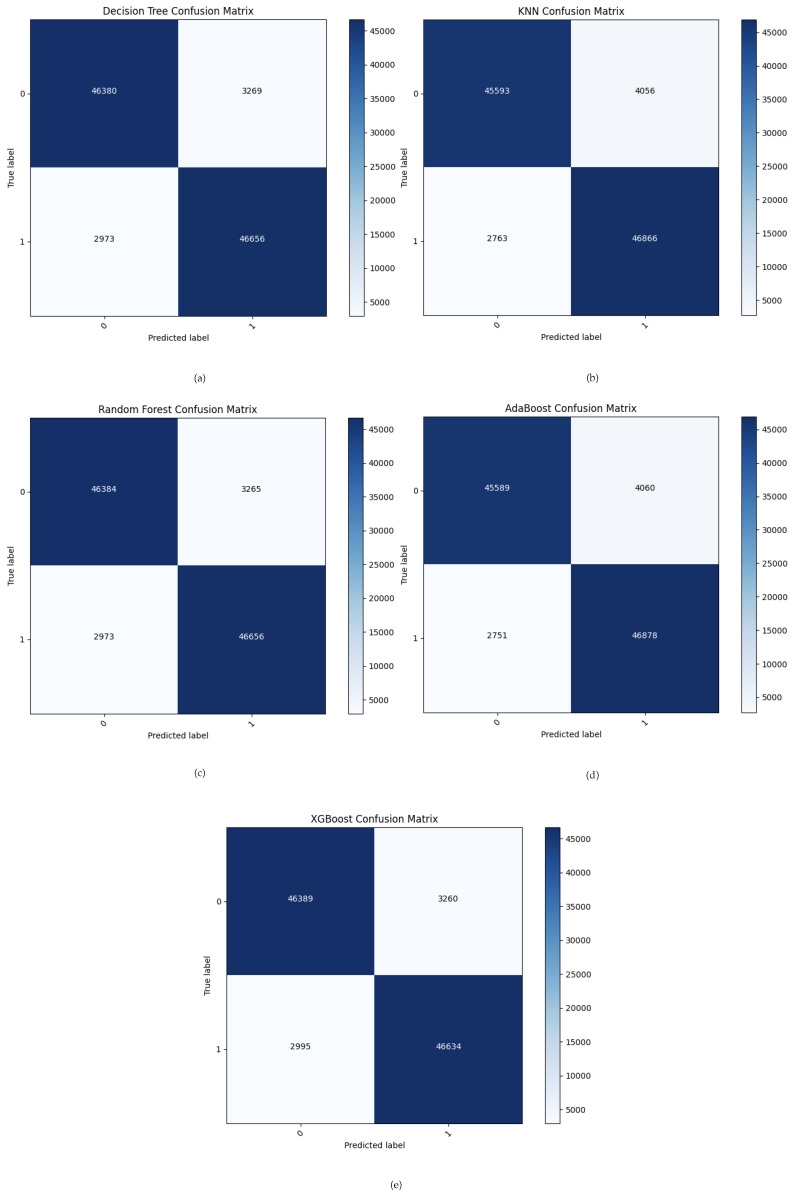
(**a**) The confusion matrix for DT. (**b**) The confusion matrix for KNN. (**c**) The confusion matrix for RF. (**d**) The confusion matrix for AdaBoost. (**e**) The confusion matrix for XGBoost.

Figure 7 depicts the confusion metrics of all ML models used. The evaluation and classification of MQTT traffic in the proposed system as either normal or attack messages relies on confusion metrics such as TP, FP, TN, and FN. The ML classifiers are presented: DT correctly identified 46,380 instances as normal (true negatives), and 3269 instances were incorrectly classified as abnormal. It correctly predicted 46,656 instances as abnormal (true positives) and incorrectly classified 2973 instances as normal. The KNN method correctly identified 45,593 instances as negatives. In comparison, 4056 instances were incorrectly defined as abnormal, and 46,866 instances were correctly identified as positives, with 2763 instances incorrectly identified as normal. Regarding the RF method, there were 46,384 correctly identified as normal and 46,656 abnormal instances, but it incorrectly classified 3265 normal instances and 2973 abnormal instances. The AdaBoost algorithm demonstrated a performance of 45,589 true negatives and 46,878 true positives along with 4060 false positives and 2751 false negatives. XGBoost showed robust predictive ability: correctly identifying 46,389 instances as true negatives and 46,634 instances as true positives while encountering 3260 instances as false positives and 2995 instances as false negatives.

## 6. Conclusions and Future Work

In this research, we proposed an approach to securing the traffic of the MQTT protocol in IoT ecosystems. We presented a robust feature engineering and ML framework aimed at improving performance. The study explores the impact of automated feature engineering, specifically task-specific feature selection. Feature selection can lead to more effective and efficient cyber-attack classification systems for MQTT traffic. By comparing and analyzing optimal feature selection for the MQTTset dataset, we chose 10 significant features. This resulted in valuable advantages such as an improved model to get faster training and testing times, enhanced accuracy, reduced dimensionality of the feature set, decreased impact of external factors, and optimization of storage and processing resources.

Furthermore, we developed and evaluated five ML algorithms—DT, KNN, RF, AdaBoost, and XGBoost—to classify MQTT traffic as normal or abnormal. The outcome of our schemes show that it is possible to detect cyber-attacks on MQTT traffic effectively. Among these models, RF showed the highest accuracy of 0.9633, followed by DT with 0.9629 and AdaBoost with 0.9629. In addition, XGBoost achieved an accuracy of 0.9629, while KNN achieved an accuracy of 0.9627. Our study also outperformed other studies [22,25] in terms of accuracy and F1-score, as shown in Table 10. Our future work aims to explore more efficient and scalable methods in ML and deep learning techniques that can significantly optimize our outcomes.

## Figures and Tables

**Table 1:** Summary of related work.

Paper	Year	Dataset	Approach	Evaluation Metric	Attacks	MQTT Protocol
[19]	2019	MQTT dataset	XGBoost, LSTM, GRU	Accuracy, F-beta score	DoS, MitM, Intrusion	✓
[20]	2019	Artemis Own Dataset	k-Means, SO GAAL, OCSVM, RF, Isolation forest, Autoencoder	Accuracy	DoS attack	✓
[21]	2020	MQTT-IoT-IDS2020 Dataset	LR, k-NN, DT, RF, SVM, NB	Precision, Recall, F1-score	Aggressive scan, UDP scan, SSH and MQTT brute force	✓
[22]	2020	MQTTset	NN, RF, NB, DT, GB, MLP	Accuracy, F1-score	Brute force, DoS, Flood, Malformed, Slowite	✓
[6]	2021	MQTT-IoT-IDS2020	DNN, RF, DT, LSTM, NB, GRUs, KNN	Accuracy, Precision, Recall, F1-measure	MitM, Intrusion, DoS	✓
[23]	2022	Kaggle MQTT Dataset	RF, KNN, SVM	Accuracy, F1 score	DoS, Brute force	✓
[24]	2022	MQTTset	NN, RF, NB, DT, GB, MLP, CatBoost	Accuracy, Precision, Recall, F measure	Brute force, DoS, Flood, Malformed, Slowite	✓
[16]	2022	SEN-MQTTset	LR, DT, SVM, NB, KNN, GB, RF	Accuracy, F1-score, MCC, FAR, ROC, ADR, APV, NPV	Normal, Subscriber attack, Broker attack	✓
[25]	2023	MQTTset	DT, RF, NB, MLP, Bagging, AdaBoost, HistGradientboost, XGB Classifier, Stacking	Accuracy, F1-score, MCC	Brute force, DoS, Flood, Malformed, Slowite	✓
[26]	2023	IoT-MQTT dataset	Adaboost, DT, RF, XGBoost, LR, SVM	Accuracy	DoS, MitM, Intrusion	✓

**Table 2:** Comparison of our proposal with recently proposed models.

Paper	Year	Dataset	ML Models	Data Balancing	Feature Selection	Hyperparameter Tuning	Cross-Validation	Accuracy
[22]	2020	MQTTset	+	+	−	−	−	0.9159
[25]	2023	MQTTset	+	+	−	−	−	0.9538
Proposed Model	2023	MQTTset	+	+	+	+	+	0.9633

**Table 3:** The distribution of classes within the MQTTset dataset.

Dataset	Types of Attacks	Count of Packets
MQTTset	Legitimate	165,463
	DoS	130,223
	Brute force	14,501
	Malformed	10,924
	SlowITe	9202
	Flood	613

**Table 4:** Final set after selecting features.

No.	Name	Description	Protocol
1	tcp.flags	TCP flags	TCP
2	tcp.time_delta	Time TCP stream	TCP
3	tcp.len	TCP Segment Len	TCP
4	mqtt.dupflag	DUP Flag	MQTT
5	mqtt.hdrflags	Header Flags	MQTT
6	mqtt.len	Msg Len	MQTT
7	mqtt.msg	Message	MQTT
8	mqtt.msgid	Message Identifier	MQTT
9	mqtt.qos	QoS Level	MQTT
10	mqtt.conack.flags	Acknowledge Flags	MQTT

**Table 5:** Summary of the optimized hyperparameters.

Algorithm	Hyperparameter
DT	Best Hyperparameters: {‘criterion’: ‘gini’, ‘max_depth’: None, ‘min_samples_leaf’: 1, ‘min_samples_split’: 2}
KNN	Best Hyperparameters: {‘n_neighbors’: 9, ‘p’: 2, ‘weights’: ‘distance’}
RF	Best Hyperparameters: {‘max_depth’: None, ‘min_samples_leaf’: 2, ‘min_samples_split’: 5, ‘n_estimators’: 50}
AdaBoost	Best Hyperparameters: {‘base_estimator’: None, ‘learning_rate’: 1.0, ‘n_estimators’: 100}
XGBoost	Best Hyperparameters: {‘learning_rate’: 0.2, ‘max_depth’: 6, ‘min_child_weight’: 1, ‘n_estimators’: 50, ‘subsample’: 0.9}

**Table 6:** Results when using 33 features.

Features	33				
**Methods**	**DT**	**KNN**	**RF**	**AdaBoost**	**XGBoost**
Accuracy	0.9629	0.9626	0.9629	0.9629	0.9627
Precision	0.9639	0.9635	0.9641	0.9635	0.9638
Recall	0.9629	0.9626	0.9629	0.9629	0.9627
F1-Score	0.9629	0.9625	0.9630	0.9629	0.9627
ROC	0.9846	0.9564	0.9844	0.9843	0.9847
Train Time (s)	0.1716	0.01446	4.3124	4.5184	0.4979
Test Time (s)	0.01242	9.2972	0.7502	0.5319	0.0118

**Table 7:** Results when using 20 features.

Features	20				
**Methods**	**DT**	**KNN**	**RF**	**AdaBoost**	**XGBoost**
Accuracy	0.9629	0.9626	0.9629	0.9629	0.9627
Precision	0.9639	0.9635	0.9641	0.9635	0.9638
Recall	0.9629	0.9626	0.9629	0.9629	0.9627
F1-Score	0.9629	0.9625	0.9629	0.9629	0.9627
ROC	0.9846	0.9564	0.9849	0.9843	0.9847
Train Time (s)	0.1387	0.0114	4.2242	3.9348	0.4027
Test time (s)	0.0087	6.72237	0.18963	0.49958	0.0135

**Table 8:** Results when using 15 features.

Features	15				
**Methods**	**DT**	**KNN**	**RF**	**AdaBoost**	**XGBoost**
Accuracy	0.9629	0.9625	0.9629	0.9629	0.9627
Precision	0.9639	0.9634	0.9639	0.9635	0.9638
Recall	0.9629	0.9623	0.9629	0.9629	0.9627
F1-Score	0.9629	0.9624	0.9629	0.9629	0.9627
ROC	0.9846	0.9519	0.9841	0.9843	0.9847
Train Time (s)	0.1328	0.1011	4.1028	3.6642	0.4285
Test Time (s)	0.0080	29.1211	0.1911	0.4741	0.0117

**Table 9:** Results when using 10 features.

Features	10				
**Methods**	**DT**	**KNN**	**RF**	**AdaBoost**	**XGBoost**
Accuracy	0.9629	0.9627	0.9633	0.9629	0.9629
Precision	0.9639	0.9636	0.9639	0.9635	0.9639
Recall	0.9629	0.9626	0.9629	0.9629	0.9627
F1-Score	0.9629	0.9623	0.9632	0.9629	0.9629
ROC	0.9846	0.9568	0.9841	0.9843	0.9847
Train Time (s)	0.1209	0.0808	4.0888	3.1877	0.3574
Test Time (s)	0.0085	14.8136	0.1686	0.4603	0.0115

**Table 10:** Comparison of recently proposed models with our models using the same dataset.

**Paper**	**[22]**				
**Year**	**2020**				
**Features**	**33**				
**Method**	**DT**	**KNN**	**RF**	**AdaBoost**	**XGBoost**
Accuracy	0.9159	-	0.9159	-	-
Precision	-	-	-	-	-
Recall	-	-	-	-	-
F1-Score	0.9140	-	0.9140	-	-
ROC	-	-	-	-	-
Train Time (s)	148.81	-	2298.27	-	-
Test Time (s)	2.30	-	125.85	-	-
**Paper**	**[25]**				
**Year**	**2023**				
**Features**	**31**				
**Method**	**DT**	**KNN**	**RF**	**AdaBoost**	**XGBoost**
Accuracy	0.9455	-	0.9538	0.9538	0.9538
Precision	-	-	-	-	-
Recall	-	-	-	-	-
F1-Score	0.9454	-	0.9537	0.9537	0.9537
ROC	-	-	-	-	-
Train Time (s)	0.29	-	9.70	17.05	5.83
Test Time (s)	0.01	-	0.81	1.93	0.12
**Paper**	**Proposed Model**				
**Year**	**2023**				
**Features**	**10**				
**Method**	**DT**	**KNN**	**RF**	**AdaBoost**	**XGBoost**
Accuracy	0.9629	0.9627	0.9633	0.9629	0.9629
Precision	0.9639	0.9636	0.9639	0.9635	0.9639
Recall	0.9629	0.9626	0.9629	0.9629	0.9627
F1-Score	0.9629	0.9623	0.9632	0.9629	0.9629
ROC	0.9846	0.9568	0.9841	0.9843	0.9847
Train time (s)	0.1209	0.0808	4.0888	3.1877	0.3574
Test time (s)	0.0085	14.8136	0.1686	0.4603	0.0115

## Data Availability

Data available upon request.

## References

[1] Mishra B., Kertesz A. (2020). The use of MQTT in M2M and IoT systems: A survey. IEEE Access.

[2] Azzedin F., Alhejri I. A Layered Taxonomy of Internet of Things Attacks. Proceedings of the 6th International Conference on Future Networks & Distributed Systems.

[3] Khazane H., Ridouani M., Salahdine F., Kaabouch N. (2024). A Holistic Review of Machine Learning Adversarial Attacks in IoT Networks. Future Internet.

[4] Chee K.O., Ge M., Bai G., Kim D.D. (2024). IoTSecSim: A framework for modelling and simulation of security in Internet of things. Comput. Secur..

[5] Al-Masri E., Kalyanam K.R., Batts J., Kim J., Singh S., Vo T., Yan C. (2020). Investigating messaging protocols for the internet of things (IoT). IEEE Access.

[6] Khan M.A., Khan M.A., Jan S.U., Ahmad J., Jamal S.S., Shah A.A., Pitropakis N., Buchanan W.J. (2021). A deep learning-based intrusion detection system for MQTT enabled IoT. Sensors.

[7] Sánchez Lasheras F., Comminiello D., Krzemień A. (2019). Advances in complex systems and their applications to cybersecurity. Complexity.

[8] Luo C., Tan Z., Min G., Gan J., Shi W., Tian Z. (2020). A novel web attack detection system for the internet of things via ensemble classification. IEEE Trans. Ind. Inform..

[9] Schizas N., Karras A., Karras C., Sioutas S. (2022). TinyML for Ultra-Low Power AI and Large Scale IoT Deployments: A Systematic Review. Future Internet.

[10] Jaw E., Wang X. (2021). Feature selection and ensemble-based intrusion detection system: An efficient and comprehensive approach. Symmetry.

[11] Dang Q.V. Studying machine learning techniques for intrusion detection systems. Proceedings of the Future Data and Security Engineering: 6th International Conference, FDSE 2019.

[12] Hussain F., Hussain R., Hassan S.A., Hossain E. (2020). Machine Learning in IoT Security: Current Solutions and Future Challenges. IEEE Commun. Surv. Tutorials.

[13] Rupa Devi T., Badugu S. (2020). A review on network intrusion detection system using machine learning. Advances in Decision Sciences, Image Processing, Security and Computer Vision: International Conference on Emerging Trends in Engineering (ICETE).

[14] Wong H., Luo T. Man-in-the-middle attacks on MQTT-based IoT using BERT-based adversarial message generation. Proceedings of the KDD’20 Workshops: The 3rd International Workshop on Artificial Intelligence of Things (AIoT).

[15] Nasir M., Javed A.R., Tariq M.A., Asim M., Baker T. (2022). Feature engineering and deep learning-based intrusion detection framework for securing edge IoT. J. Supercomput..

[16] Siddharthan H., Deepa T., Chandhar P. (2022). Senmqtt-set: An intelligent intrusion detection in iot-mqtt networks using ensemble multi cascade features. IEEE Access.

[17] Kamaldeep M.M., Dutta M. (2023). Feature Engineering and Machine Learning Framework for DDoS Attack Detection in the Standardized Internet of Things. IEEE Internet Things J..

[18] Panda M., Mousa A.A.A., Hassanien A.E. (2021). Developing an Efficient Feature Engineering and Machine Learning Model for Detecting IoT-Botnet Cyber Attacks. IEEE Access.

[19] Alaiz-Moreton H., Aveleira-Mata J., Ondicol-Garcia J., Muñoz-Castañeda A.L., García I., Benavides C. (2019). Multiclass classification procedure for detecting attacks on MQTT-IoT protocol. Complexity.

[20] Ciklabakkal E., Donmez A., Erdemir M., Suren E., Yilmaz M.K., Angin P. Artemis: An intrusion detection system for MQTT attacks in the internet of things. Proceedings of the 38th Symposium on Reliable Distributed Systems (SRDS).

[21] Hindy H., Bayne E., Bures M., Atkinson R., Tachtatzis C., Bellekens X. (2020). Machine learning-based IoT intrusion detection system: A MQTT case study (MQTT-IoT-IDS2020 dataset). International Networking Conference.

[22] Vaccari I., Chiola G., Aiello M., Mongelli M., Cambiaso E. (2020). MQTTset, a new dataset for machine learning techniques on MQTT. Sensors.

[23] Makhija J., Shetty A.A., Bangera A. (2022). Classification of attacks on MQTT-based IoT system using machine learning techniques. International Conference on Innovative Computing and Communications.

[24] Vijayan P.M., Sundar S. (2022). An Efficient CatBoost Classifier Approach to Detect Intrusions in MQTT Protocol for Internet of Things. International Conference on Computational Intelligence and Data Engineering.

[25] Zeghida H., Boulaiche M., Chikh R. (2023). Securing MQTT protocol for IoT environment using IDS based on ensemble learning. Int. J. Inf. Secur..

[26] Muñoz Castañeda Á.L., Mata J.A.A., Aláiz-Moretón H. (2023). Characterization of threats in IoT from an MQTT protocol-oriented dataset. Complex Intell. Syst..

[27] Wood A.D., Stankovic J.A. (2002). Denial of service in sensor networks. Computer.

[28] Hwang H.C., Park J., Shon J.G. (2016). Design and implementation of a reliable message transmission system based on MQTT protocol in IoT. Wirel. Pers. Commun..

[29] Atilgan E., Ozcelik I., Yolacan E.N. MQTT Security at a Glance. Proceedings of the 2021 International Conference on Information Security and Cryptology (ISCTURKEY).

[30] Morelli U., Vaccari I., Ranise S., Cambiaso E. DoS Attacks in Available MQTT Implementations: Investigating the Impact on Brokers and Devices, and supported Anti-DoS Protections. Proceedings of the ARES 2021: The 16th International Conference on Availability, Reliability and Security.

[31] Shirodkar S.A. (2023). Brute Force Attacks Detection on IoT Networks using Deep Learning Techniques. Int. J. Adv. Res. Sci. Commun. Technol..

[32] Qaddoori S.L., Ali Q.I. (2023). An Efficient Security Model for Industrial Internet of Things (IIoT) System Based on Machine Learning Principles. Rafidain Eng. J..

[33] Vaccari I., Aiello M., Cambiaso E. (2020). SlowITe, a Novel Denial of Service Attack Affecting MQTT. Sensors.

[34] Al-Omari M., Rawashdeh M., Qutaishat F., Alshira H.M., Ababneh N. (2021). An intelligent tree-based intrusion detection model for cyber security. J. Netw. Syst. Manag..

[35] Sinsomboonthong S. (2022). Performance Comparison of New Adjusted Min-Max with Decimal Scaling and Statistical Column Normalization Methods for Artificial Neural Network Classification. Int. J. Math. Math. Sci..

[36] Arya L., Gupta G.P. Ensemble Filter-based Feature Selection Model for Cyber Attack Detection in Industrial Internet of Things. Proceedings of the 2023 9th International Conference on Advanced Computing and Communication Systems (ICACCS).

[37] Dhal S.B., Jungbluth K., Lin R., Sabahi S.P., Bagavathiannan M., Braga-Neto U., Kalafatis S. (2022). A Machine-Learning-Based IoT System for Optimizing Nutrient Supply in Commercial Aquaponic Operations. Sensors.

[38] Li X., Chen W., Zhang Q., Wu L. (2020). Building auto-encoder intrusion detection system based on random forest feature selection. Comput. Secur..

[39] Khan H., Haq I.U., Munsif M., Mustaqeem, Khan S.U., Lee M.Y. (2022). Automated wheat diseases classification framework using advanced machine learning technique. Agriculture.

[40] Kumar P., Gupta G.P., Tripathi R. (2021). An ensemble learning and fog-cloud architecture-driven cyber-attack detection framework for IoMT networks. Comput. Commun..

[41] Gad A.R., Nashat A.A., Barkat T.M. (2021). Intrusion Detection System Using Machine Learning for Vehicular Ad Hoc Networks Based on ToN-IoT Dataset. IEEE Access.

[42] Danso P.K., Neto E.C.P., Dadkhah S., Zohourian A., Molyneaux H., Ghorbani A.A. Ensemble-based Intrusion Detection for Internet of Things Devices. Proceedings of the 2022 IEEE 19th International Conference on Smart Communities: Improving Quality of Life Using ICT, IoT and AI (HONET).

[43] Cvitić I., Peraković D., Periša M., Gupta B. (2021). Ensemble machine learning approach for classification of IoT devices in smart home. Int. J. Mach. Learn. Cybern..

[44] Pannakkong W., Thiwa-Anont K., Singthong K., Parthanadee P., Buddhakulsomsiri J. (2022). Hyperparameter tuning of machine learning algorithms using response surface methodology: A case study of ANN, SVM, and DBN. Math. Probl. Eng..

[45] Ghori K.M., Abbasi R.A., Awais M., Imran M., Ullah A., Szathmary L. (2019). Performance analysis of different types of machine learning classifiers for non-technical loss detection. IEEE Access.

[46] Boateng E.Y., Otoo J., Abaye D.A. (2020). Basic tenets of classification algorithms K-nearest-neighbor, support vector machine, random forest and neural network: A review. J. Data Anal. Inf. Process..

[47] Zebari R., Abdulazeez A., Zeebaree D., Zebari D., Saeed J. (2020). A comprehensive review of dimensionality reduction techniques for feature selection and feature extraction. J. Appl. Sci. Technol. Trends.

